# Activation of TLR7 and Innate Immunity as an Efficient Method Against COVID-19 Pandemic: Imiquimod as a Potential Therapy

**DOI:** 10.3389/fimmu.2020.01373

**Published:** 2020-06-11

**Authors:** Konstantinos Poulas, Konstantinos Farsalinos, Charilaos Zanidis

**Affiliations:** Laboratory of Molecular Biology and Immunology, Department of Pharmacy, University of Patras, Patras, Greece

**Keywords:** Imiquimod (IMQ), innate immunity, COVID-19, SARS - CoV-2, immune activation

Coronaviruses are a large group of viruses that can cause illness, the symptoms of which are ranging from the common cold to more severe diseases, like Middle East Respiratory Syndrome (MERS) and Severe Acute Respiratory Syndrome (SARS). The 2019 novel coronavirus, called “SARS-CoV-2” is a new strain that causes Corona Virus Disease 2019 (COVID-19), for which no effective treatment has been found until now. The outbreak of SARS-CoV-2, that first emerged in Wuhan in December 2019, has rapidly spread throughout the world (1, 2). Considering the ongoing outbreak in China and the rapid global spread of COVID-19, contaminated with SARS-CoV-2, it contributed to the World Health Organization (WHO) declaration of Public Health Emergency on 30th January 2020 (3). A total of more than 5,000,000 laboratory-confirmed cases were reported worldwide as of May 22nd, 2020.

According to numerous publications the patients tended to have lymphopenia, higher infection-related biomarkers and several elevated inflammatory cytokines [i.e., tumor necrosis factor alpha (TNF-α), interleukins IL-2R and IL-6]. The total number of B cells, T cells and Natural Killer (NK) cells is significantly decreased in patients with COVID-19 and is more evident in the severe cases, compared to the non-severe group. T cells were proved to be more affected by SARS-CoV-2 as T cell count was nearly half the lower reference limit. The function of CD4+, CD8+ T cells, and NK cells was within normal range and no significant difference was found between severe cases and non-severe ones (4). Higher serum levels of cytokines TNF-α, IL-1, and IL-6 and chemokine IL-8 were found in patients with severe COVID-19 compared to individuals with mild disease (4).

The first line of protection against viral infection is a rapid and well-coordinated innate immune response, but when the immune response is dysregulated, it can result in excessive inflammation, even death (5). Qin et al. demonstrated pronounced lymphopenia and low counts of CD3+ and CD4+ cells in COVID-19 cases (4).

It is well-known that in early life, when the adaptive functions of the immune system are still underdeveloped, the innate immune system—the non-specific immune response—is really important. The main aim of the innate immune system is to prevent the further spread of any foreign pathogen. It functions by starting a signaling cascade after the recognition of what is called “pathogen-associated molecular patterns.” The pattern recognition receptors (PRRs) are responsible for this cascade (6). For RNA viruses, especially, it is known that the Toll-like receptors (TLRs)—TLRs 3, 7, and 8—are the really important PRRs (7). The innate immune system is sensitive in detecting potentially pathogenic foreign material, and by this is activating downstream signaling to eventually induce transcription factors in the nucleus, that in turn promote the synthesis and release of types I and III IFNs and a number of other important pro-inflammatory cytokines. A second round of signaling ensures that any infected cells and all the surrounding uninfected, are starting to express a great number of interferon-stimulated genes that further establish the so-called antiviral state (8).

Adaptive—specific/acquired—immune system is the second line of defense, communicating, however, with the innate one. Strict distinction between these two systems and the response caused is not accurate. In the respiratory tract, several cell types and immune mechanisms are very important for this defense and express aspects from both of immunity. NKs, T cells, mucosal-associated invariant T cells, and neutrophils, form a bridge between the innate and adaptive machineries and play very important roles during the clearance of respiratory viruses (8).

Imiquimod (IQ), member of the imidazoquinolines family, is a well-studied molecule and the only one currently approved for clinical use, which has been proved to enhance both the non-specific and specific immune response, and in particular the cell-mediated pathways (9). IQ is the first small molecule disclosed to act through TLR activation, especially TLR7. Preclinical and clinical experiments have proved strong antiviral and antitumor properties. IQ is able to modify the immune response, by inducing the expression and production of a number of cytokines. These cytokines are further stimulating T cells. As a result, IQ can enhance innate and acquired cellular immunity (10).

As for the innate immune system, IQ is able to induce IFNa, IL-6, and IL-12 and TNFa. IQ is stimulating NK cells activity; macrophages are also activated and by this way are secreting cytokines and nitric oxide. B lymphocytes are induced to start proliferation and differentiation (11). IQ, by influencing the innate immunity, has proved its great potential to combat and treat viral infections. The cellular arm of the two pathways in the acquired immune response is induced by Imiquimod, although this is not a direct effect.

IQ, by activating innate immunity is able to indirectly stimulate and activate the cellular arm of the immune response and the production of the T-helper type 1 (Th1) cytokine IFNγ. In parallel, IQ suppresses the humoral arm of acquired immunity, by inhibiting the expression of Th2 cytokines (e.g., IL4 and IL5) (11). IQ is further modifying the immune response by activating the Langerhans' cells (10). These cells are migrating, by IQ stimulation, to the regional lymph nodes, enhancing antigen presentation to T cells (9).

Topical IQ as a 5% cream (Aldara) is approved for the treatment of genital/perianal warts. IQ is listed as a Category C drug, as for safety. When applied topically its half-life is ~30 h. IQ is well-accepted (when applied locally), safe and with limited adverse effects (12). We propose, however, the repurposing/repositioning of IQ and the systematic administration, by compounding suppositories, containing 6.25 mg each. There is a small number of published studies proposing/explaining the systematic administration of the drug for its antiviral activity against HPV and HIV (13, 14). We have clear evidence that IQ is able to offer satisfactory stimulation of innate and acquired immunity, helping the elimination of SARS-CoV-2, at least during the early phases of infection. We propose the trial of IQ as a potential anti-SARS-CoV-2 drug.

## Author Contributions

The concept is based on an idea by KP and KF. CZ and KP drafted the manuscript. All authors contributed to the article and approved the submitted version.

## Conflict of Interest

The authors declare that the research was conducted in the absence of any commercial or financial relationships that could be construed as a potential conflict of interest.
